# Supplementary material for: The causal relationship between the human gut microbiota and pyogenic arthritis: a Mendelian randomization study
Source: Front Cell Infect Microbiol. 2024 Nov 26;14:1452480. doi: 10.3389/fcimb.2024.1452480 (PMC11629706; doi:10.3389/fcimb.2024.1452480)
Supplement: Supplementary file 1 [file DataSheet1.zip › mendelian test/ebi-a-GCST90027605.csv_leave_one_out.pdf]

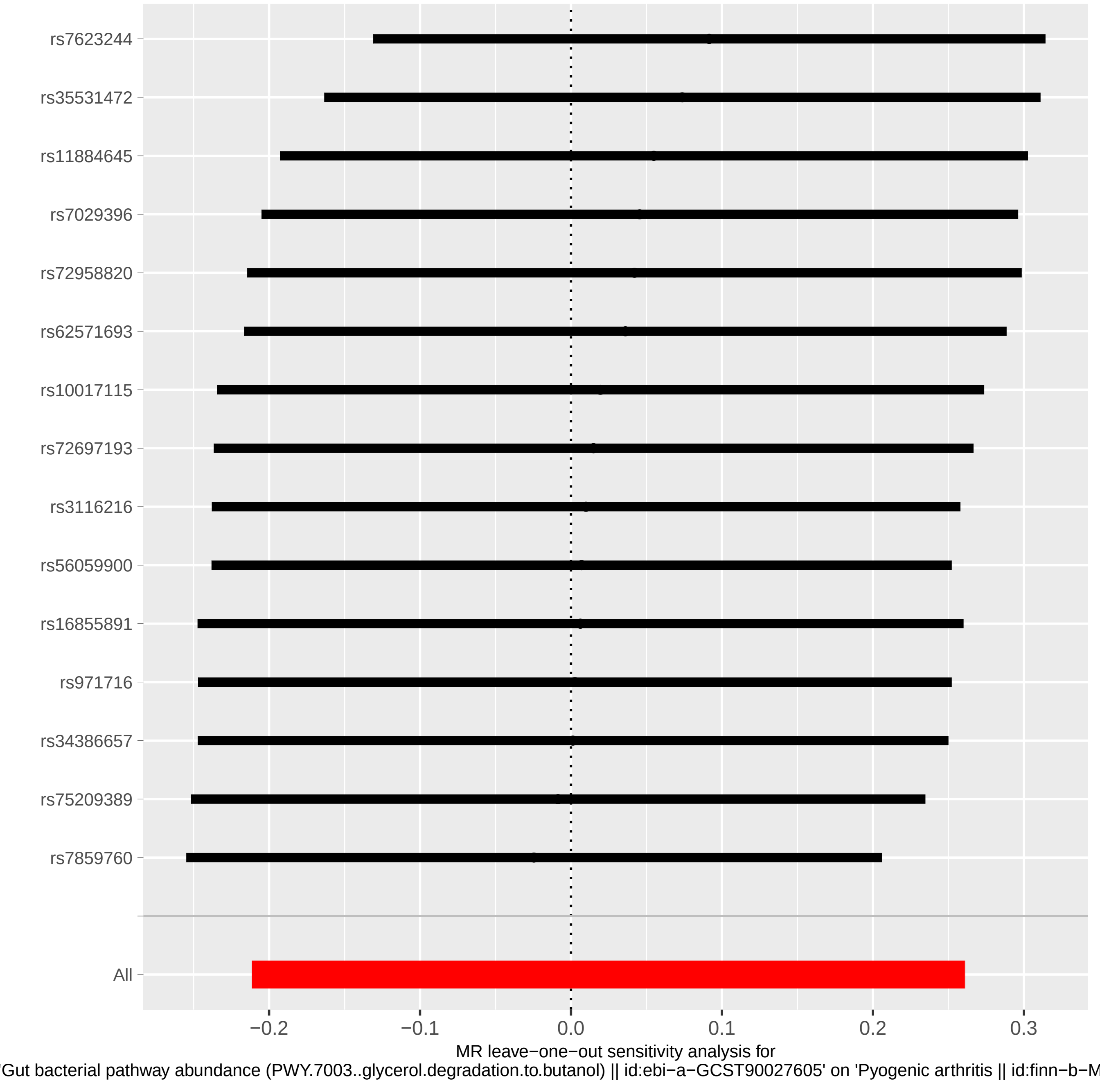

rs7623244
rs35531472
rs11884645
rs7029396
rs72958820
rs62571693
rs10017115
rs72697193
rs3116216
rs56059900
rs16855891
rs971716
rs34386657
rs75209389
rs7859760
All
−0.2
−0.1
0.0
0.1
0.2
0.3
MR leave−one−out sensitivity analysis for
'Gut bacterial pathway abundance (PWY.7003..glycerol.degradation.to.butanol) || id:ebi−a−GCST90027605' on 'Pyogenic arthritis || id:finn−b−M
